# Comparison of Duplex and Quadruplex Folding Structure Adenosine Aptamers for Carbon Nanotube Field Effect Transistor Aptasensors

**DOI:** 10.3390/nano11092280

**Published:** 2021-09-02

**Authors:** Hong Phan T. Nguyen, Thanihaichelvan Murugathas, Natalie O. V. Plank

**Affiliations:** 1 School of Chemical and Physical Sciences, Victoria University of Wellington, Wellington 6021, New Zealand; Hongphan.Nguyen@vuw.ac.nz; 2 The MacDiarmid Institute for Advanced Materials and Nanotechnolgy, Victoria University of Wellington, Wellington 6021, New Zealand; 3 Department of Physics, University of Jaffna, Jaffna 40000, Sri Lanka; thanihai@univ.jfn.ac.lk

**Keywords:** aptasensor, carbon nanotube FET, adenosine, adenosine detection, aptamer, biosensor

## Abstract

Carbon nanotube field effect transistor (CNT FET) aptasensors have been investigated for the detection of adenosine using two different aptamer sequences, a 35-mer and a 27-mer. We found limits of detection for adenosine of 100 pM and 320 nM for the 35-mer and 27-mer aptamers, with dissociation constants of 1.2 nM and 160 nM, respectively. Upon analyte recognition the 35-mer adenosine aptamer adopts a compact G-quadruplex structure while the 27-mer adenosine aptamer changes to a folded duplex. Using the CNT FET aptasensor platform adenosine could be detected with high sensitivity over the range of 100 pM to 10 µM, highlighting the suitability of the CNT FET aptasensor platform for high performance adenosine detection. The aptamer restructuring format is critical for high sensitivity with the G-quadraplex aptasensor having a 130-fold smaller dissociation constant than the duplex forming aptasensor.

## 1. Introduction

Carbon nanotube field-effect transistor aptasensors have been demonstrated as an effective semiconducting platform for sensor applications in clinical health diagnostics [1] for the detection of a variety of targets, including small molecules [2,3], proteins [4,5], and metal ions [6]. Aptamers have been developed as the primary recognition element in biosensors due to their high specificity and affinity, reproducibility, and small size [7,8]. Aptamers for specific targets are synthesized using the systematic evolution of ligands by exponential enrichment (SELEX) in vitro and depending on how the target is prepared and bound, aptamers with different lengths and structures will be selected [9,10]. However, the selection of optimized aptamers for electronic biosensing in body fluids can be challenging for many reasons, including the need to overcome Debye screening in biological fluids and optimize the aptamer morphology to maximize the selectivity sensitivity [11,12]. In the presence of the target analyte, aptamers are known to adopt a folded 3-D conformation structure, and these conformational changes of the aptamers are important for the development of biosensors. The ability of aptamers to make conformational changes can be extremely sensitive to the surrounding environment including ionic strength, pH, temperature, and even metal ions on electrode surfaces [13,14]. For effective CNT FET aptasensors, the major conformal changes of the aptamer must take place as close to the CNT channel as possible, e.g., within the Debye length [6,15].

Adenosine plays an essential and complex role in human physiological function, with impacts in coronary blood flow, tumour immunity, and the development of neurological diseases [16,17,18] to name a few. Adenosine can also promote the survival of cancerous tumours by inhibiting the cell-mediated anti-tumour immune response [19]. Monitoring adenosine levels could be clinically useful for vasodilation, blood pressure control and antiarrhythmic treatments [20], with the potential of real time monitoring of adenosine in bio-fluids in the diagnosing and monitoring of cancer patients. Considering the potential impact of accurate adenosine detection in these clinical settings, the development of a simple, accurate and sensitive adenosine sensor is highly desirable. Despite the clear motivation for adenosine sensors, its detection remains a challenge, mainly because of the low levels found in biological samples. The adenosine level in plasma from healthy people was from 13 ± 7 nM [21] and significantly increased in patients with cardiogenic shock to 2.74 ± 1.03 µM and heart failure 1.33 ± 0.27 µM [22]. Numerous methods and platforms have been developed for detection of adenosine, including colorimetric [23,24], fluorescence [25,26], and cyclic voltammetry [27,28] on silver nanoparticles [23], graphene oxide [25], gold [27], and CNTs [28] (see Table S1 for a full summary). Although some of these sensors have shown the sensitivity required for clinical adenosine detection, sensitivity in a single sensor over a wide range of adenosine levels and the need of real-time detection are problems that remain to be solved.

In this study, we create CNT FET aptasensors for detecting adenosine molecules using two different aptamer sequences, a 27-mer and a 35-mer which are known to adopt duplex [23] and G-quadruplex structures [29], respectively. Both the 27-mer and 35-mer adenosine aptamers tested here have previously detected low concentrations of adenosine (21 nM [23] and 5 µM [29], respectively), which are considerably lower than the several hundred µM concentrations that previous work with different adenosine aptamer sequences achieved [30,31]. These aptamers have also shown excellent selectivity in previous studies. The 27-mer duplex aptamer based sensor showed an improved selectivity to adenosine against control molecules guanin, thymine, urea, L-Lysine, DL-Methionine, and L-Threonin [23]. The 35-mer aptamer, which forms the G-quadruplex structure upon binding to the adenosine molecule was reportedly detecting adenosine molecules with high selectivity when compared to control molecules, cytidine triphosphate, guanosine triphosphate, and uridine triphosphate [29]. There has been no direct comparison between the 27-mer and the 35-mer adenosine aptamers on the same sensing platform, with the G-quadruplex forming 35-mer having only been used in one previous study for adenosine detection [29]. Here we have found limits of detection for adenosine of 320 nM for the 27-mer aptamer and 100 pM for the 35-mer aptamer on nominally identical sensing platforms. Our results show unambiguously that the effect of the conformational changes of aptamers are important for improved sensitivity in CNT FET aptasensor design.

## 2. Materials and Methods

### 2.1. Carbon Nanotube Field-Effect Transistor Fabrication

Thin films of CNTs were fabricated directly onto SiO_2_/Si substrates using a solution deposition method [2,32,33]. The CNT suspensions were made by ultrasonication of a tweezer tip amount of CNT bucky paper (NanoIntegris Isonantube S-99) in 10 mL of 1,2-dichlorobenzene (DCB) (Sigma Aldrich, St. Louis, MO, USA) for 30 min. SiO_2_/Si substrates were cleaned in acetone, IPA and then dried in a stream of nitrogen. A 10 mg/mL solution of 2-mercaptopyridine (99%, Sigma Aldrich, St. Louis, MO, USA) in ethanol was then drop-cast over the entire SiO_2_/Si surface and left for 20 min before rinsing the substrates in ethanol for 2 s to remove excess solvent. The device chips were submerged into the CNT-DCB suspension for 2 h and rinsed in ethanol solution for 10 min. The CNT films were then selectively etched in an oxygen plasma before deposition of Cr (5 nm)/Au (50 nm) source and drain electrodes, with channels of 40 µm length and 100 µm width. The electrodes were then encapsulated with photoresist AZ1518 (Microchemicals, Newton, MA, USA) and hard baked at 200 °C for 10 min, creating an open space with dimensions of 10 µm length and 100 µm width in the CNT FET channel to the environment.

### 2.2. Aptamer Functionalisation

Two different sequences of adenosine aptamers, the 27-mer and 35-mer (sequences listed in Table 1) were used as the aptasensor receptors. All aptamers were functionalised onto the CNT side walls using 1-pyrenebutanoic acid succinimidyl ester (PBASE) as a molecular linker [34]. The fabricated CNT FETs were submerged into 1 mM PBASE (95%, Sigma Aldrich, St. Louis, MO, USA) in methanol solution for 1 h, then rinsed a few times in pure methanol to remove excess PBASE and washed in DI water for 5 s. Meanwhile, the aptamers were diluted to a concentration of 1 µM aptamer solution in 20 mM Tris-HCl buffer (Sigma Aldrich, St. Louis, MO, USA) and then denatured at 70 °C for 5 min in an oven. A total of 100 µL of the prepared aptamer solution was added onto the channel of the CNT FET at room temperature overnight in a closed petri dish. Finally, the unbound aptamers on the surface were removed by washing the devices with 20 mM Tris buffer and DI water before drying in N_2_.

### 2.3. Electrical Characterisation

**Figure 1:**
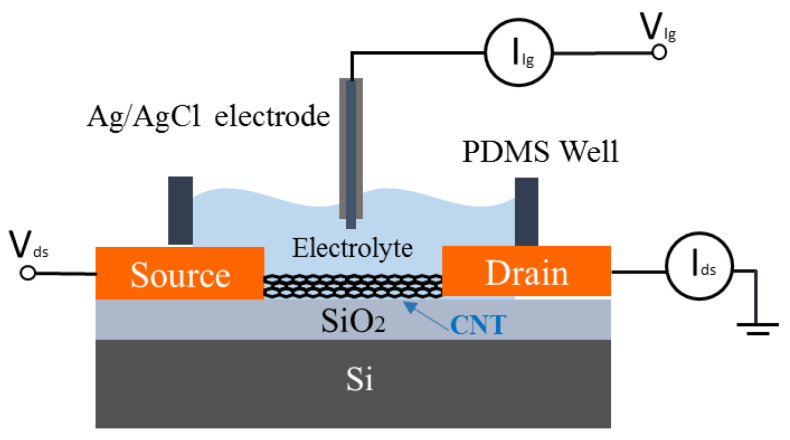
Schematic of the experimental setup for the electrical characterisation of a CNT FET and aptamer-functionalized CNT FET biosensors.

The electrical characterisation of the CNT FETs was carried out with an Agilent 4156C parameter analyser (Agilent Technologies, Santa Clara, CA, USA) connected to the CNT FETs via micromanipulators and a Rucker and Kolls probes station. Figure 1 shows a schematic of the electrical characterisation setup of CNT FET aptasensors using liquid-gated geometry. The source/drain electrodes are connected to the parameter analyser using micromanipulators. A polydimethylsiloxane (PDMS) well was used to keep the electrolyte on the channel and a gate voltage was applied via an Ag/AgCl reference electrode. We have selected a 2 mM tris buffer for these experiments as optimised in our previous work on potassium detection [6]. For the CNT conductance measurement, the liquid-gate voltage (Vlg) was swept from −0.5 V to +1 V while the source-drain voltage (Vds) was set at 100 mV. For real-time electrical aptasensor measurements, V_lg_ was set at 0 V, and V_ds_ is set at 100 mV. The readings were recorded at the interval of 1 s. Initially, 100 µL of 2 mM Tris-HCl buffer was added into PDMS well as an electrolyte for liquid measurements. As the first test, another 10 µL of the 2 mM Tris-HCl buffer solution was added into the well. After the initial buffer addition of 10 µL buffer, adenosine solutions were added to the PDMS well every 500 s.

## 3. Results

### 3.1. Characteristics of CNT FET Aptasensors

**Figure 2:**
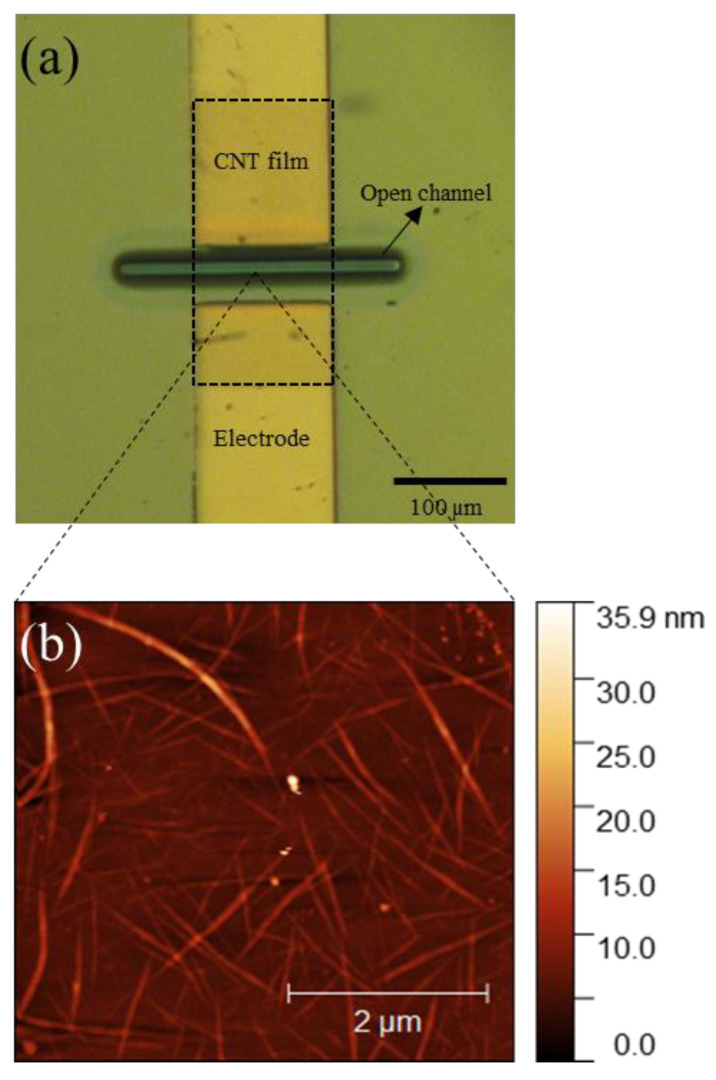
(**a**) Microscopic image of the CNT channel with encapsulated source and drain electrodes in a completed FET device and (**b**) AFM image of the CNT film deposited on the SiO_2_/Si substrate which consist of large bundles with a diameter of ~20 nm and single tubes.

Figure 2a shows the optical microscope image of the encapsulated channel of one of the CNT FETs fabricated. Only a 10 µm length by 100 µm width part of the channel is open to the environment and the electrodes and the CNT-metal interface is completely encapsulated by the photoresist layer. The encapsulation was applied to electrically isolate the source-drain electrodes and the Schottky junctions at the metal-CNT interface from the gate electrolyte [6,15]. Figure 2b shows an atomic force microscopy (AFM) image of the CNT channel deposited on the SiO_2_/Si substrates and confirms the presence of large CNT bundles as well as smaller bundles or isolated CNTs, similar to our previously reported work [15,35]. The average lengths of the CNTs were measured as 2.14 µm and the CNT bundle diameter were found to be around 10–20 nm similar to our previous work [15]. All the CNT devices used in this study were fabricated under similar conditions, allowing us to minimise the device-to-device variation. The transfer characteristics of the CNT FETs were measured in 2 mM Tris buffer before and after the immobilisation of both the adenosine aptamers as shown in Figure S1. The results confirmed the immobilization of aptamers on the CNT channel.

### 3.2. Sensing Response

**Figure 3:**
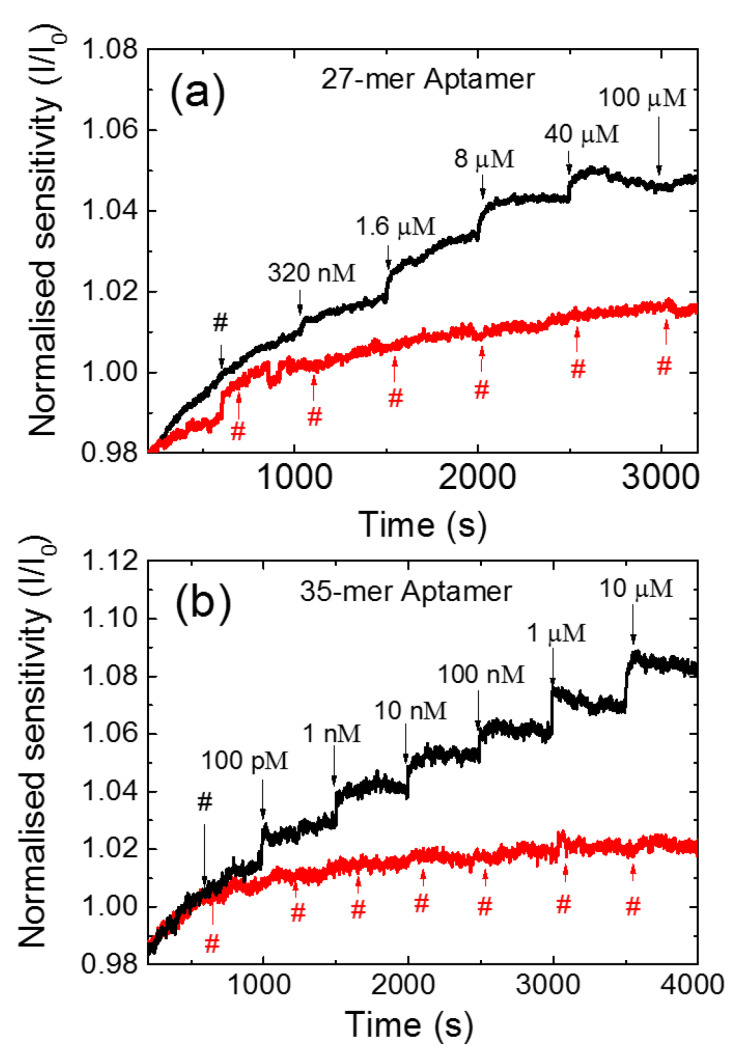
The normalized current response to adenosine for (**a**) the 27-mer adenosine aptamer and (**b**) the 35-mer adenosine aptamer (black line) immobilised CNT FET aptasensors; and control measurement with successive addition of 2 mM Tris-HCl buffer (red line).

The sensing measurements of the CNT FET aptasensors were carried out with Vlg = 0 V and Vds = 100 mV while recording the current at 1 s intervals. A 2 mM Tris- HCl was chosen as the buffer, as the 10 nm Debye length of the buffer is close to the fully extended length of both the aptamers used in this study, 9 nm and 11 nm for the 27 and 35-mer respectively. Figure 3 shows the current responses for (a) the 27-mer and (b) the 35-mer aptasensors as adenosine was added to the PDMS well at intervals of 500 s (black line). As a control, we have also measured the response of 2 mM Tris-HCl buffer added to the PDMS well of a similarly prepared CNT FET aptasensor at the same time intervals (red line). Each measurement started with the initial load of 110 µL of 2 mM Tris-HCl buffer in the PDMS well for 1000 s. The adenosine solution was then added at volumes between 10 and 25 µM for every 500 s in successively greater concentrations, which resulted in the total concentration in the PDMS well increasing from 320 nM to 100 µM for 27-mer adenosine aptamer and 100 pM to 10 µM for 35-mer adenosine aptamer (taking into account the concentration in the PDMS well prior to each addition). The same protocol of analyte addition was performed for the 27-mer and 35-mer. The different range of 320 nM to 100 µM for 27-mer adenosine aptamer was chosen as the 27-mer adenosine aptasensor showed no response to adenosine concentrations of 1 nM and the signal became stable at 100 µM. The drain current increased as the adenosine concentration was increased for both aptamer sequences, which is consistent with our previous CNT FET aptasensor and attributed to electrostatic gating [6,15]. The 35-mer aptasensor showed a greater current response compared to the 27-mer aptasensor. During the sensing measurements taken between 1000 s and 3200 s (Figure 3a), the 27-mer aptasensor showed an increase in the normalised current I/I_0_ of 14%. The 35-mer aptasensor showed a normalised current I/I_0_ increase of 26% over the same time interval (Figure 3b). The detection limit of the adenosine level achieved was 320 nM and 100 pM for the 27-mer and 35-mer adenosine aptamers, respectively.

The target binding dynamics of both the 35-mer and 27-mer aptasensors were then investigated by comparing the time taken to reach 90% of the maximum sensing response after adding the target (t_90_) of both the aptasensors. The t_90_ values are calculated as given by Groß, A. et al. [36]. The average t_90_ value of the 35-mer and 27-mer aptasensors were found to be 86.5 (±19.5) s, and 202.4 (±98.1) s, respectively. It is clear that the G-quadruplex forming 35-mer aptasensors showed a much faster sensing response when compared to the duplex forming 27-mer aptasensors. The faster sensing response can be attributed to the close proximity of the negative charge in the 35-mer aptamers to the dominating sensing hotspots in the CNT bundle network [15].

**Figure 4:**
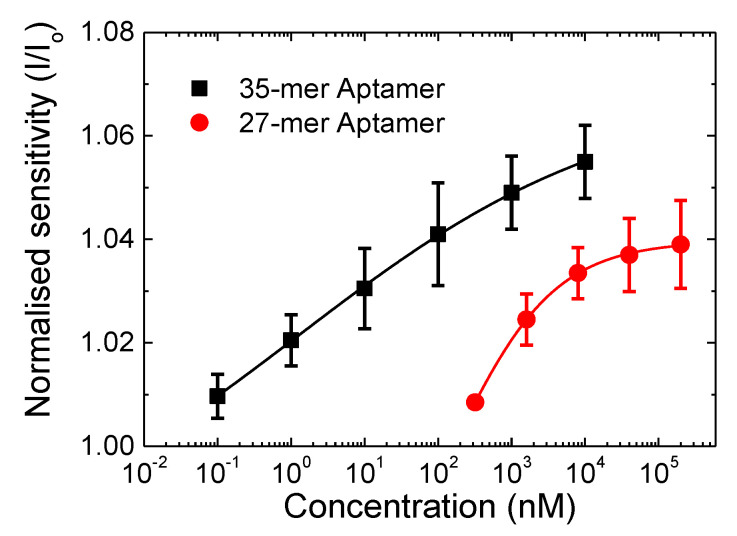
Normalised sensitivity in response to the added adenosine concentration for the 27-mer (circle) and 35-mer adenosine aptamer (square) immobilised CNT-FETs aptasensors. The lines indicate the corresponding Hill-Langmuir fittings of the sensing response.

In order to compare the sensitivity of the 27-mer aptasensor to the 35-mer aptasensor on the CNT FET platform, the normalised sensitivity response (I/I_0_) is plotted in Figure 4. The error bars are from I/I_0_ for three sensing tests from devices fabricated under the same conditions. The 35-mer adenosine aptasensor achieves a lower detection limit of 100 pM compared to 320 nM for the 27-mer adenosine aptasensor, and higher sensitivity of 1.055 compared to 1.039, respectively.

The fitting curves of the sensing responses, Figure 4, follow the Hill-Langmuir isotherm model for equilibrium binding of a ligand by a receptor [37,38] given as:

(1) $$\frac{I}{I_{0}}=A\frac{{\left(\frac{c}{K_{d}}\right)}^{n}}{1+{\left(\frac{c}{K_{d}}\right)}^{n}}+Z,$$

where *A* presents the maximum response when all binding sites are occupied, *c* is the adenosine concentration, *K_d_* is the dissociation constant, *n* is the Hill coefficient, and *Z* is an offset parameter. The best fit (R^2^ > 0.99) values of the Hill-Langmuir model for both the 27-mer and 35-mer adenosine sequences are summarized in Table 2 where the Hill coefficient value of *n* < 1 indicates a negative cooperativity in the binding of adenosine molecules to the CNT FET biosensor. Negative cooperativity represents when the binding of a ligand to a specific aptamer receptor makes it more difficult for that aptamer to then bind to other molecules, resulting in the most sensitive responses [39]. Similar negative cooperativity was also observed by graphene FET aptasensors for detection of cytokine [40] and thrombin [41], and CNT FETs to detect protein [42].

From the Hill-Langmuir isotherm model, the dissociation constant *K_d_* for the interaction between adenosine molecules and the 35-mer aptamers was 1.2 nM, which is 130-fold smaller than the 160 nM found for the 27-mer aptamers. These results are substantially lower than the first reported dissociation constant of an adenosine aptamer/adenosine complex of 6 µM [43]. Prior to our work, the lowest *K_d_* reported for an adenosine aptasensor was 400 nM [44], achieved via a triplex DNA aptamer sequence. Moreover, our 27-mer CNT FET aptasensor has a *K_d_* value of 160 nM, which is more than 20-fold stronger than the 3.7 µM reported for the same adenosine sequence using a dual-polarization interferometry-based technique [44]. Presently there are no reported values of the dissociation constant for the 35-mer aptamer. The choice of the 27-mer and 35-mer aptamers used here was based on a literature survey where these sequences have been shown to have excellent limit of detection, up to 21 nM [23,45,46] and 5 μM [29], respectively, however there are no reported *K_d_* values for comparison.

## 4. Discussion

There are several possibilities that could cause the differences in *K_d_* between the 27-mer and 35-mer aptamers tested here and those in the literature and we need to be cautious when interpreting this data. The sensing platform in our experiments is a CNT FET, which is ultrasensitive to charge modulation close to the CNT surface and within the Debye limit. Furthermore, our CNT film is not flat and uniform, the morphology of the device includes various junctions and conduction paths. This means that the sensitivity of the sensor is not purely governed by the CNT-aptamer-analyte interactions in the same way as other platforms and CNT junctions may be playing a key role in enhancing the aptasensor sensitivity [15]. Moreover, the variation between the choice of buffer solutions and pH value between our work (2 mM Tris-HCl, pH 7.4), and those used by other teams (Table S1), can also significantly affect the aptamer binding constants. Finally, the actual nucleotide sequences for adenosine detection used in this work are different from most of those used in the other experiments (see Table S1), which should also have a significant impact on the resulting *K_d_* and sensor performance.

**Figure 5:**
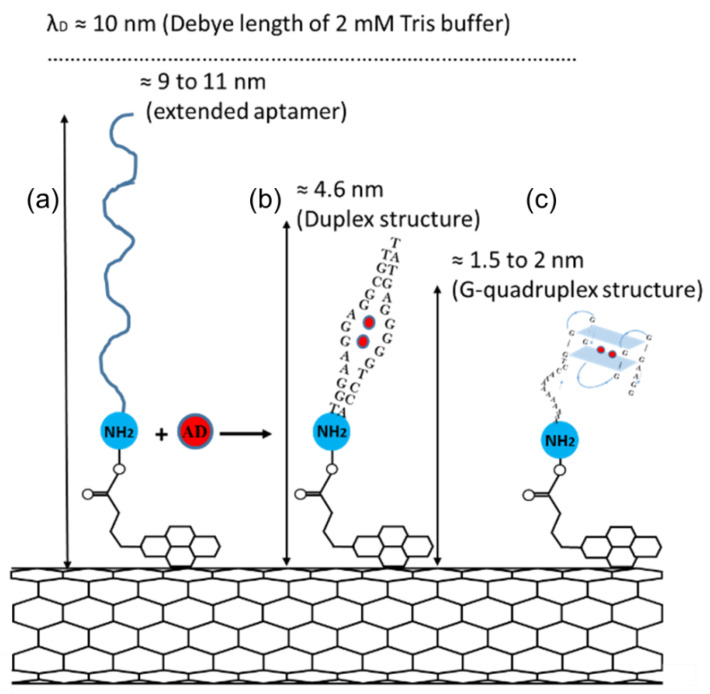
Schematic representation of (**a**) fully extended adenosine aptamers immobilized onto the surface of a CNT via PBASE linker and (**b**) the formation of Duplex and (**c**) G-quadruplex structure by 27-mer and 35-mer aptamers, respectively, after adenosine molecule binding (not to scale).

The different sensitivity of the CNT FET 27-mer and 35-mer aptasensors can be explained by considering the conformational change of aptamers after adenosine exposure. Figure 5 schematically illustrates the conformation structure of both the aptamers upon binding with adenosine molecules. The 27-mer adenosine aptamer forms a duplex structure [23,45,46], whereas the 35-mer aptamer forms a G-quadruplex structure [29].

The structures formed can have a huge influence on the expected sensitivity of the sensors. The CNT-FET aptasensors are sensitive to any changes in the charge distribution occurring within the Debye length [6,15,47], which within our sensing set up, with 2 mM Tris buffer, is 10 nm [6]. If the aptamers were to be extended to the full length of their chemical chains, they would reach to 9 nm and 11 nm for the 27-mer and 35-mer aptamers, respectively. When folded, the G-quadruplex structure has been reported around 1.5 nm to 2 nm [48,49] and the duplex structure is expected to be folded in half to a height of around 4.5 nm. Both conformation changes of the adenosine aptamers bring the negatively charged DNA backbone closer to the CNT wall and alter the charge distribution in the Debye length region of the device [4,50], acting as a molecular gate [4,6]. The G-quadruplex forming 35-mer aptamer with a shorter folding length of 2 nm compared to the 4.5 nm of the 27-mer aptamer duplex, shows a more effective and faster sensing response. Moreover, when compared to the duplex, the G-quadruplex aptamer has twice the negative charge density and a higher electrostatic potential per unit length [51] which could be a reason for the faster sensing response. The 35-mer can also detect adenosine at concentrations as low as 100 pM compared to 320 nM for the 27-mer aptamer, indicating that the 35-mer has a stronger binding affinity and is overall more effective as a sensor receptor. Further work with other sensing platforms is required to verify the improved binding affinity.

We can compare our adenosine detection performance with other types of adenosine aptasensors, as shown in Table S1. Our CNT FET aptasensors show significant sensitivity in the presence of 100 pM adenosine using 35-mer aptamer. This detection level is comparatively lower than most of previously reported adenosine detection and is within the biologically relevant testing regime for clinical biosensors. The adenosine concentration in plasma is 13 ± 7 nM from healthy people [21], however it increases significantly to a few micromolar in patients with heart failure or cardiogenic shock [22]. Recently Y. Wang et al. [52] and Das et al. [53] achieved a 0.02 pM and 1 pM adenosine detection limit respectively. These limit detections were lower than our results, however, the detection range of these sensors, from 0.05 pM to 17 pM for the gold electrode platform [52] and 1 pM to 10 nM for the CNT FET platform [53], are lower than the full range of the biological adenosine detection level for unwell patients. Compared to the adenosine aptasensors in Table S1, the sensitivity of our CNT FET aptasensors with these aptamers have exhibited multiple advantages. First, our detection limits of 100 pM up to 10–100 µM is within the biological range of interest for adenosine in human blood. Second, since the real-time measurement produces an immediate sensing signal, our CNT FET platform could be useful for clinical diagnosis and monitoring applications. Third, the CNT FET aptasensors are easy to fabricate, label-free, with high sensitivity and that can meet the needs of point of care applications.

## 5. Conclusions

The aptamer folding structurers are a crucial factor for improved sensitivity of the CNT FET aptasensors. We have shown that the sensitivity of CNT FET aptasensors for adenosine was higher for the 35-mer G-quadruplex aptamer in comparison to the 27-mer duplex aptamer. The 35-mer G-quadruplex aptamer functionalized CNT FET showed a clear increase in current over the range 100 pM to 10 µM with a level of detection of 100 pM compared to 320 nM for 27-mer adenosine aptamer. Both aptamers also show an improved binding affinity in comparison to previous studies, with dissociation constants of 1.2 nM for the 35-mer and 160 nM for the 27-mer adenosine aptamer by applying the Hill-Langmuir binding equation. We have demonstrated that the CNT FET aptasensor platform is a viable candidate for adenosine detection and has performed better than numerous other sensor platforms and aptamer systems in the literature.

## Figures and Tables

**Table 1:** DNA sequences used in this study.

Adenosine Aptamer	Sequence
27-mer	5′-NH2-ACCTGGGGGAGTATTGCGGAGGAAGGT-3′
35-mer	5′-NH2-AAAAAAAAAACCTGGGGGAGTATTGCGGAGGAAGG-3′

**Table 2:** Summary of the best-fit parameters from the Langmuir-Hill isotherm for the 35-mer and 27-mer adenosine aptasensors.

Aptamer Sequence	A	Kd	N	Z
35-mer	0.09 ± 0.015	(1.2 ± 1.08) × 10^−9^ M	0.2 ± 0.03	0.97 ± 0.01
27-mer	0.07 ± 0.02	(1.6 ± 1.4) × 10^−7^ M	0.6 ± 0.08	0.96 ± 0.02

## Supplementary Materials

The following are available online at https://www.mdpi.com/article/10.3390/nano11092280/s1, Figure S1: Transfer characteristics of the (a) 27-mer and (b) 35-mer adenosine aptamers modified CNT FET (Source-drain voltage was kept at 100 mV for all measurements), Table S1: Comparison of adenosine detection in various aptasensors platforms.
