# Supplementary material for: Comparison of Duplex and Quadruplex Folding Structure Adenosine Aptamers for Carbon Nanotube Field Effect Transistor Aptasensors
Source: Nanomaterials (Basel). 2021 Sep 2;11(9):2280. doi: 10.3390/nano11092280 (PMC8468449; doi:10.3390/nano11092280)
Supplement: Supplementary file 1 [file nanomaterials-11-02280-s001.zip › nanomaterials-1341044-supplementary.pdf]

nanomaterials

MDPI

Article

Comparison of Duplex and Quadruplex Folding Structure Adenosine Aptamers for Carbon Nanotube Field Effect Transistor Aptasensors

Hong Phan T. Nguyen [1,2], Thanihaichelvan Murugathas [3] and Natalie O. V. Plank [1,2,*]

1 School of Chemical and Physical Sciences, Victoria University of Wellington, Wellington 6021, New Zealand; Hongphan.Nguyen@vuw.ac.nz
2 The MacDiarmid Institute for Advanced Materials and Nanotechnolgy, Victoria University of Wellington, Wellington 6021, New Zealand
3 Department of Physics, University of Jaffna, Jaffna 40000, Sri Lanka; thanihai@univ.jfn.ac.lk
* Correspondence: Natalie.Plank@vuw.ac.nz; Tel.: +64-4-463-5031

1. Characteristics of CNT FET Aptasensors

The transfer characteristics of the CNT FETs were measured in 2 mM Tris- HCl buffer before and after the immobilisation of the adenosine aptamers as shown in Fig.S1 (a, b). All pristine CNT network FETs showed an ambipolar transfer curve with a positive threshold voltage, which indicates a hole dominated electrical conduction across the channel at zero gate bias [1,2]. The immobilisation of both of the adenosine aptamers resulted a positive shift in threshold voltage and an increase in the in the device current in the ON state. The positive shift in threshold voltage indicates the tethering of negatively changed aptamers on the CNT channel [3,4].

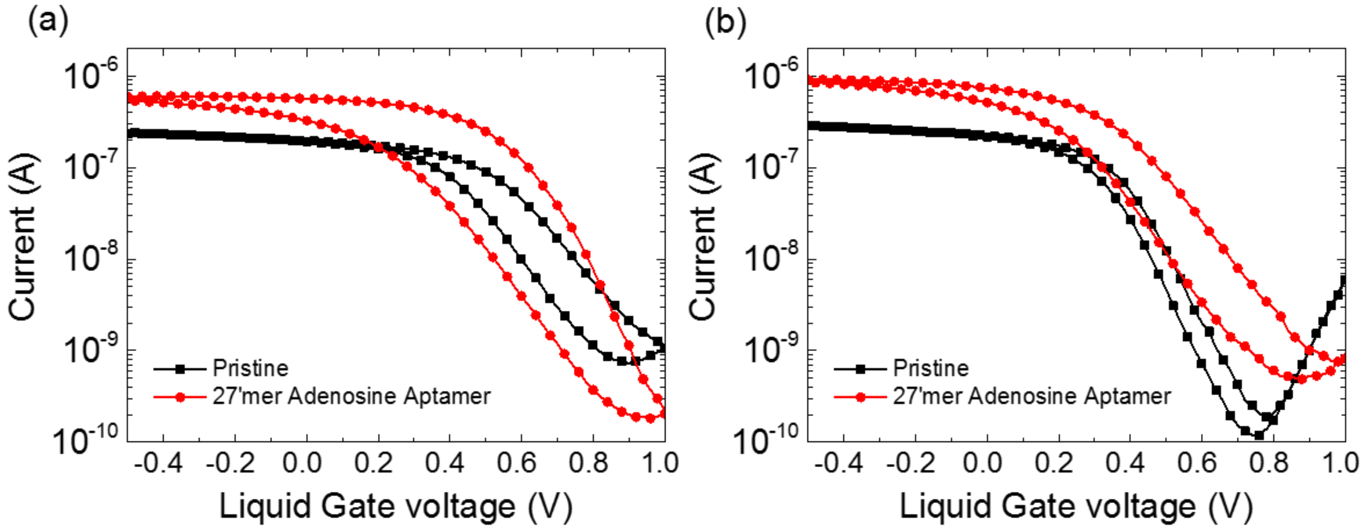

Figure S1. Transfer characteristics of the (**a**) 27-mer and (**b**) 35-mer adenosine aptamers modified CNT FET (Source-drain voltage was kept at 100 mV for all measurements).

2. Comparison of Adenosine Detection in Various Aptasensors Platforms and Detection Methods

Table S1. Comparison of adenosine detection in various aptasensors platforms.

Platform	Detection Method	Detection Range	LOD	K_d	Ref
Silicon Oxynitride Surface	Dual-Polarization Interferometry	0.25 – 15 µM	0.14 µM – 0.32 µM	3.7 µM *	[5]
Gold	Alternating Current Voltammetry (ACV)	0.1 nM – 100 µM	90.8 pM	Not stated*	[6]
Gold	Cyclic Voltammetry & Electrical Impedance Spectroscopy	0.05 pM – 17 pM	0.02 pM	Not stated	[7]
Gold	Cyclic Voltammetry and Chronocoulometry	0.05 nM – 20 nM	32 pM	Not stated	[8]
AgNPs	UV-vis spectroscopy/ Colorimetric	60 – 280 nM	21 nM	Not stated *	[9]
PAA Nano- channels	Circular dichroism Spectroscopy	0.05 – 10 mM	5 µM	Not stated **	[10]
Not Stated	Fluorescence	50 nM – 2 µM	50 nM	400 nM	[11]
rGO-AuNCs	Fluorescence	0.1 nM – 1 mM	100 pM	Not stated	[12]
Graphene Oxide	Fluorescence	0 nM – 320 nM	2.4 nM	Not stated	[13]
AgNCs – CdTe-QDs	Photocurrent	1 fM – 10 nM	0.5 fM	Not stated	[14]
Exo III	Colorimetric	50 nM – 6 µM	17 nM	Not stated	[15]
Cu-AgNCs	Fluorescence	0 – 1 µ M	19 nM	Not stated	[16]
SWCNT FETs	Chemiresistance/current	1 pM – 10 nM	1 pM	Not stated	[17]
MWCNTs-IL-CHIT	Cyclic Voltammetry & Electrical Impedance Spectroscopy	500 pM – 400 nM	150 pM	Not stated	[18]
CNT FETs	Transistor Current	100 pM – 10 µM	100 pM	1.2 nM **	This work
CNT FETs	Transistor Current	320 nM – 100 µM	320 nM	160 nM *	This work

* Used the same 27-mer aptamer as this work. ** Used the same 35-mer aptamer as this work.

Note that the remaining studies used a variety of different aptamer sequences.

rGO – AuNCs : Reduced Graphene Oxide and Gold Nanoclusters.

AgNCs_CdTe_QDs: Silver nanoparticles-assisted ion-exchange reaction with CdTe quantum dots.

MWCNTs-IL-CHIT: Multi-walled carbon nanotubes – Ionic liquid – Chitosan

References

1. Murugathas, T.; Zheng, H.Y.; Colbert, D.; Kralicek, A. V.; Carraher, C.; Plank, N.O.V. Biosensing with Insect Odorant Receptor Nanodiscs and Carbon Nanotube Field-Effect Transistors. *ACS Appl. Mater. Interfaces* **2019**, *11*, 9530–9538.
2. Heller, I.; Janssens, A.M.; Männik, J.; Minot, E.D.; Lemay, S.G.; Dekker, C. Identifying the Mechanism of Biosensing with Carbon Nanotube Transistors. *Nano Lett.* **2008**, *8*, 591–595.
3. Zheng, H.Y.; Alsager, O.A.; Zhu, B.; Travas-Sejdic, J.; Hodgkiss, J.M.; Plank, N.O.V. V Electrostatic Gating in Carbon Nanotube Aptasensors. *Nanoscale* **2016**, *8*, 13659–13668.

4. Thanihaichelvan, M.; Browning, L.A.; Dierkes, M.P.; Reyes, R.M.; Kralicek, A. V.; Carraher, C.; Marlow, C.A.; Plank, N.O.V. Metallic-Semiconducting Junctions Create Sensing Hot-Spots in Carbon Nanotube FET Aptasensors near Percolation. *Biosens. Bioelectron.* **2018**, *130*, 408–413.
5. HU, T.; YANG, F.; YANG, X.R. Real-Time Study of Interaction Between Adenosine Triphosphate and Its Aptamer Using Dual Polarization Interferometry. *Chinese J. Anal. Chem.* **2017**, *45*, 944–950.
6. Cui, L.; Lu, M.; Li, Y.; Tang, B.; Zhang, C. yang A Reusable Ratiometric Electrochemical Biosensor on the Basis of the Binding of Methylene Blue to DNA with Alternating AT Base Sequence for Sensitive Detection of Adenosine. *Biosens. Bioelectron.* **2018**, *102*, 87–93.
7. Wang, Y.; Feng, J.; Tan, Z.; Wang, H. Electrochemical Impedance Spectroscopy Aptasensor for Ultrasensitive Detection of Adenosine with Dual Backfillers. *Biosens. Bioelectron.* **2014**, *60*, 218–223.
8. Shen, J.; Wang, H.; Li, C.; Zhao, Y.; Yu, X.; Luo, X. Label-Free Electrochemical Aptasensor for Adenosine Detection Based on Cascade Signal Amplification Strategy. *Biosens. Bioelectron.* **2017**, *90*, 356–362.
9. Yousefi, S.; Saraji, M. Optical Aptasensor Based on Silver Nanoparticles for the Colorimetric Detection of Adenosine. *Spectrochim. Acta - Part A Mol. Biomol. Spectrosc.* **2019**, *213*, 1–5.
10. Yu, J.; Zhang, L.; Xu, X.; Liu, S. Quantitative Detection of Potassium Ions and Adenosine Triphosphate via a Nanochannel-Based Electrochemical Platform Coupled with G-Quadruplex Aptamers. *Anal. Chem.* **2014**, *86*, 10741–10748.
11. Patel, M.; Dutta, A.; Huang, H. A Selective Adenosine Sensor Derived from a Triplex DNA Aptamer. *Anal. Bioanal. Chem.* **2011**, *400*, 3035–3040.
12. Zhang, D.; Ma, J.; Meng, X.; Xu, Z.; Zhang, J.; Fang, Y.; Guo, Y. Electrochemical Aptamer-Based Microsensor for Real-Time Monitoring of Adenosine in Vivo. *Anal. Chim. Acta* **2019**, *1076*, 55–63.
13. You, J.; You, Z.; Xu, X.; Ji, J.; Lu, T.; Xia, Y.; Wang, L.; Zhang, L.; Du, S. A Split Aptamer-Labeled Ratiometric Fluorescent Biosensor for Specific Detection of Adenosine in Human Urine. *Microchim. Acta* **2019**, *186*.
14. Zhao, Y.; Tan, L.; Gao, X.; Jie, G.; Huang, T. Silver Nanoclusters-Assisted Ion-Exchange Reaction with CdTe Quantum Dots for Photoelectrochemical Detection of Adenosine by Target-Triggering Multiple-Cycle Amplification Strategy. *Biosens. Bioelectron.* **2018**, *110*, 239–245.
15. Xu, L.; Shen, X.; Li, B.; Zhu, C.; Zhou, X. G-Quadruplex Based Exo III-Assisted Signal Amplification Aptasensor for the Colorimetric Detection of Adenosine. *Anal. Chim. Acta* **2017**, *980*, 58–64.
16. Ahn, J.K.; Kim, H.Y.; Baek, S.; Park, H.G. A New S-Adenosylhomocysteine Hydrolase-Linked Method for Adenosine Detection Based on DNA-Templated Fluorescent Cu/Ag Nanoclusters. *Biosens. Bioelectron.* **2017**, *93*, 330–334.
17. Das, B.K.; Tlili, C.; Badhulika, S.; Cella, L.N.; Chen, W.; Mulchandani, A. Single-Walled Carbon Nanotubes Chemiresistor Aptasensors for Small Molecules: Picomolar Level Detection of Adenosine Triphosphate. *Chem. Commun.* **2011**, *47*, 3793–3795.
18. Shahdost-fard, F.; Salimi, A.; Sharifi, E.; Korani, A. Fabrication of a Highly Sensitive Adenosine Aptasensor Based on Covalent Attachment of Aptamer onto Chitosan-Carbon Nanotubes-Ionic Liquid Nanocomposite. *Biosens. Bioelectron.* **2013**, *48*, 100–107.
